# Supplementary material for: Astrocytic microdomains from mouse cortex gain molecular control over long-term information storage and memory retention
Source: Commun Biol. 2021 Oct 5;4:1152. doi: 10.1038/s42003-021-02678-x (PMC8492720; doi:10.1038/s42003-021-02678-x)
Supplement: Supplementary file 2 — Supplementary Information [file 42003_2021_2678_MOESM2_ESM.pdf]

αBDNFpro

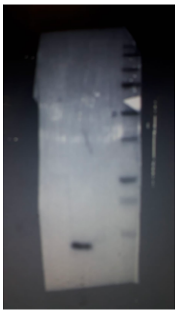

αmBDNF

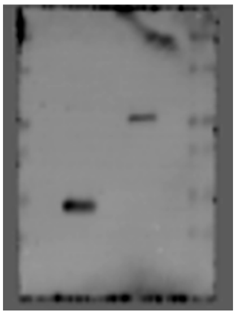

Supplementary Fig.1: Uncropped blot referring to Figure 1 a.

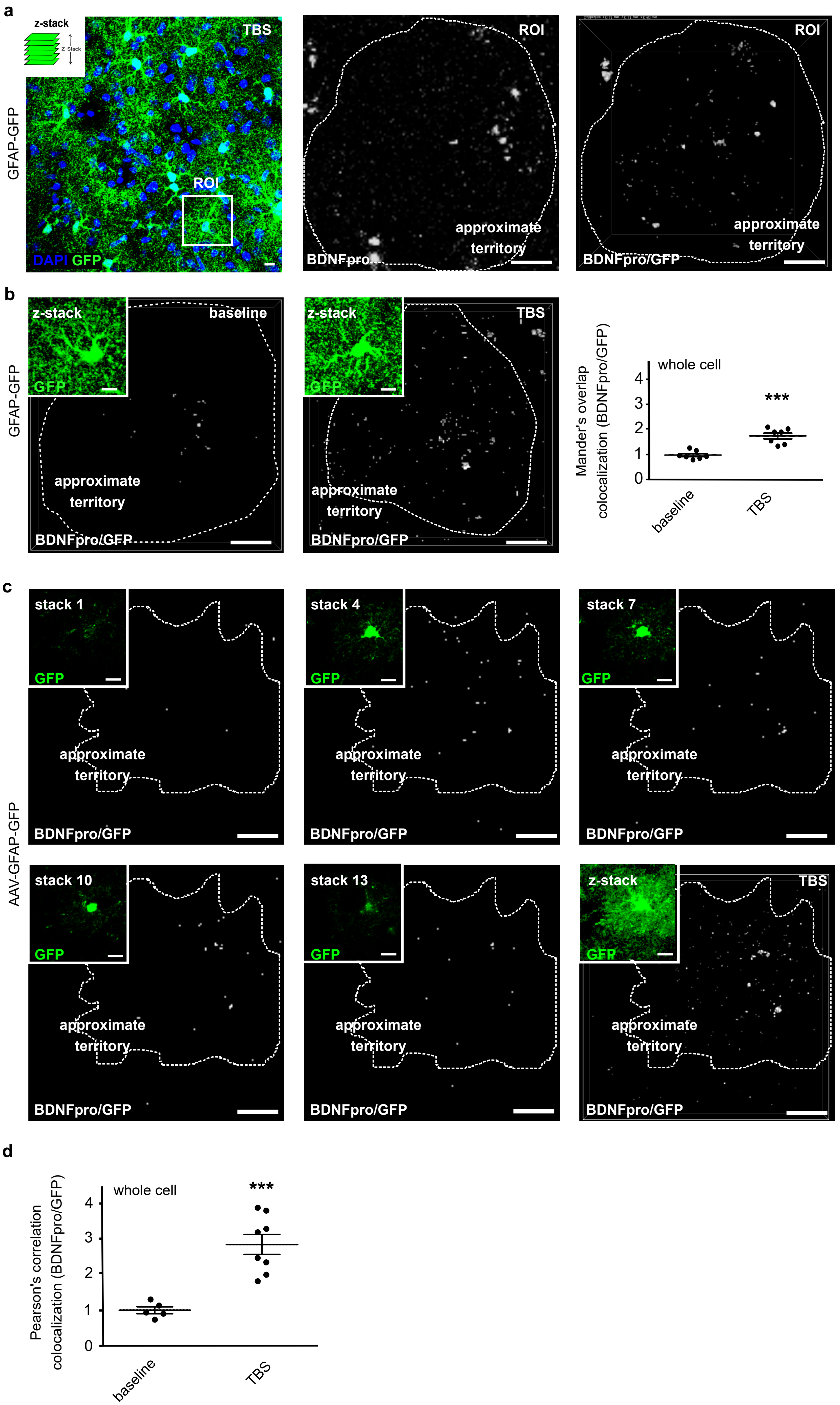
a
z-stack
GFAP-GFP
TBS
ROI
DAPI GFP
BDNFpro
approximate territory
BDNFpro/GFP
b
z-stack
baseline
GFP
approximate territory
BDNFpro/GFP
TBS
Mander's overlap colocalization (BDNFpro/GFP)
whole cell
***
0
1
2
3
4
baseline
TBS
c
AAV-GFAP-GFP
stack 1
stack 4
stack 7
stack 10
stack 13
d
Pearson's correlation colocalization (BDNFpro/GFP)

Supplementary Fig.2: BDNFpro localization in astrocytes

(a) Cortical slices from GFAP-GFP mice fixed 10 min after TBS and processed for immunostaining. z-stack reconstruction shows astrocytes labeled by GFP. Magnification of a ROI shows BDNFpro immunoreactivity and BDNFpro/GFP colocalization signal of one GFP-astrocyte delimited by an approximate territory (white dashed). Scale bars: 10 µm.

(b) z-stack reconstruction of BDNFpro/GFP colocalization signals in astrocytes from baseline- and TBS-slices from GFAP-GFP mice. Insets show GFP signal. BDNFpro/GFP colocalization was quantified in whole cell using Mander's overlap. *** $p<0.001$ (Unpaired t-test); (n = 7 cells, 4 slices, 3 mice for baseline; n = 7 cells, 4 slices, 4 mice for TBS). Scale bars: 10 µm.

(c) Stack-by-stack reconstruction of the same astrocytes shown in Fig. 1c. BDNFpro/GFP colocalization in control littermates injected with AAV-GFAP-GFP virus from stack (0,5 µm thick) 1, 4, 7, 10, and 13. The last panel shows BDNFpro/GFP colocalization signal. Insets show GFP signal. Scale bars: 10 µm.

(d) BDNFpro/GFP colocalization in control littermates injected with AAV-GFAP-GFP was quantified using Pearson's correlation. *** $p<0.001$ (Unpaired t-test); (n = 5 cells, 3 slices, 3 mice for baseline; n = 8 cells, 4 slices, 4 mice for TBS).

Data are normalized to baseline and presented as mean ± SEM.

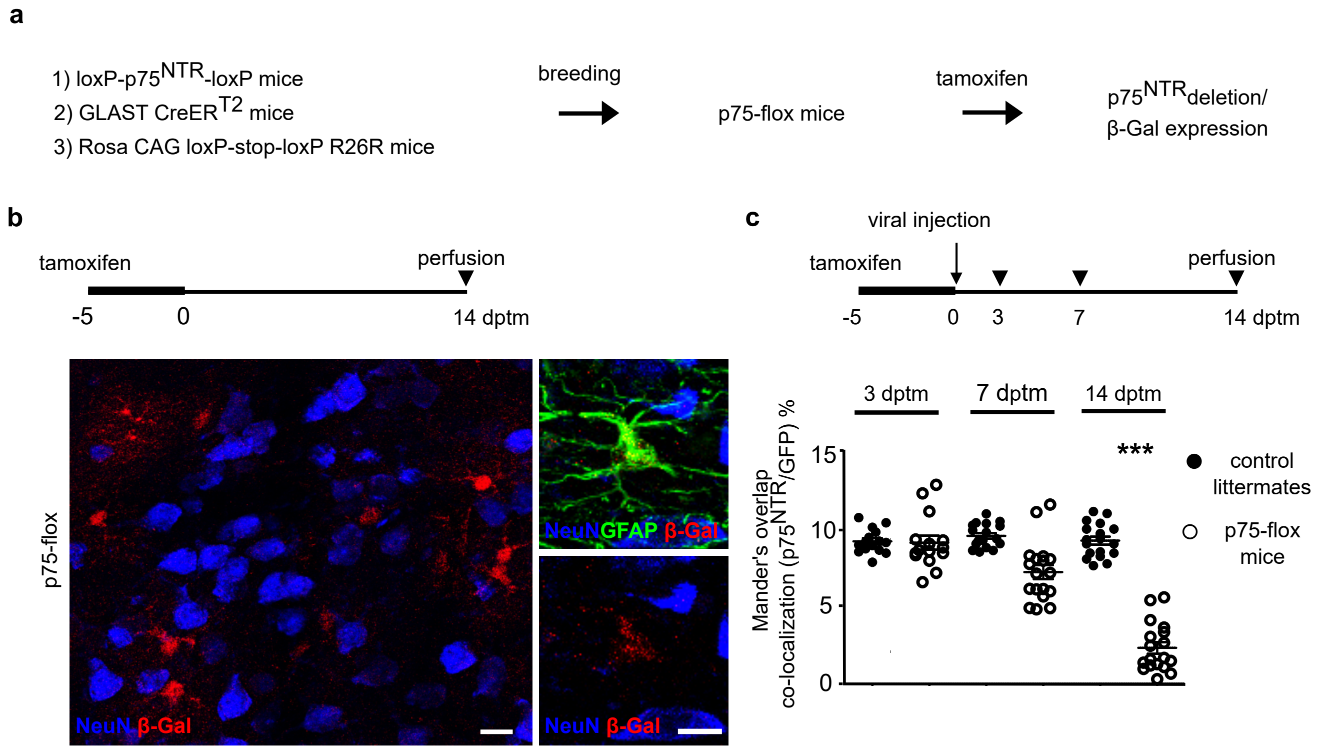

a
1) loxP-p75NTR-loxP mice
2) GLAST CreERT2 mice
3) Rosa CAG loxP-stop-loxP R26R mice
breeding
p75-flox mice
tamoxifen
p75NTR deletion/
β-Gal expression
b
tamoxifen
perfusion
-5
0
14 dptm
p75-flox
NeuN β-Gal
NeuN GFAP β-Gal
NeuN β-Gal
c
viral injection
tamoxifen
perfusion
-5
0
3
7
14 dptm
3 dptm
7 dptm
14 dptm
***
Mander's overlap
co-localization (p75NTR/GFP) %
15
10
5
0
control littermates
p75-flox mice

Supplementary Fig. 3: p75-flox mice

(a) Schematic diagram depicting the experimental paradigm. p75-flox mice were generated by crossing (1) loxP-p75^{NTR}-loxP mice, expressing flanking exons 4-6 of the p75^{NTR} gene with loxP sites; with (2) GLAST-CreER^{T2} mice, expressing the inducible version of the Cre recombinase (CreER^{T2}) under the control of the GLAST promoter and (3) Rosa-CAGloxP-stop-loxP(LSL)-R26R mice for β-Gal expression. Tamoxifen treatment in p75-flox mice causes the CreER^{T2} fusion protein to translocate into the nucleus of GLAST-expressing cells, where it recombines paired loxP sites allowing p75^{NTR} gene deletion and β-Gal expression.

(b) Schematic representation of the experimental design. Slices from p75-flox mice and control littermates treated with tamoxifen (-5 to 0) and processed for immunostaining at 14 dptm. z-stack reconstruction of a representative field shows β-Gal staining consistently excluded from NeuN labeled cells. Representative example shows one GFAP-positive astrocyte expressing β-Gal (right). Scale bars: 10 µm.

(c) Schematic representation of the experimental design. Slices from p75-flox mice and control littermates treated with tamoxifen (-5 to 0), injected with AAV-GFAP-GFP adenovirus the last day of tamoxifen treatment (0 dptm) and processed for immunostaining at 3, 7 and 14 dptm. Dot plot depicts the quantification of p75^{NTR}/GFP colocalization using Mander's overlap. Data are presented as mean ± SEM; *** $p<0.001$ (Unpaired t-test); (n = 15 cells, 4 slices, 3 mice for 3 dptm; n = 18 cells, 5 slices, 4 mice for 7 dptm; n = 18 cells, 5 slices, 4 mice for 14 dptm for each experimental group).

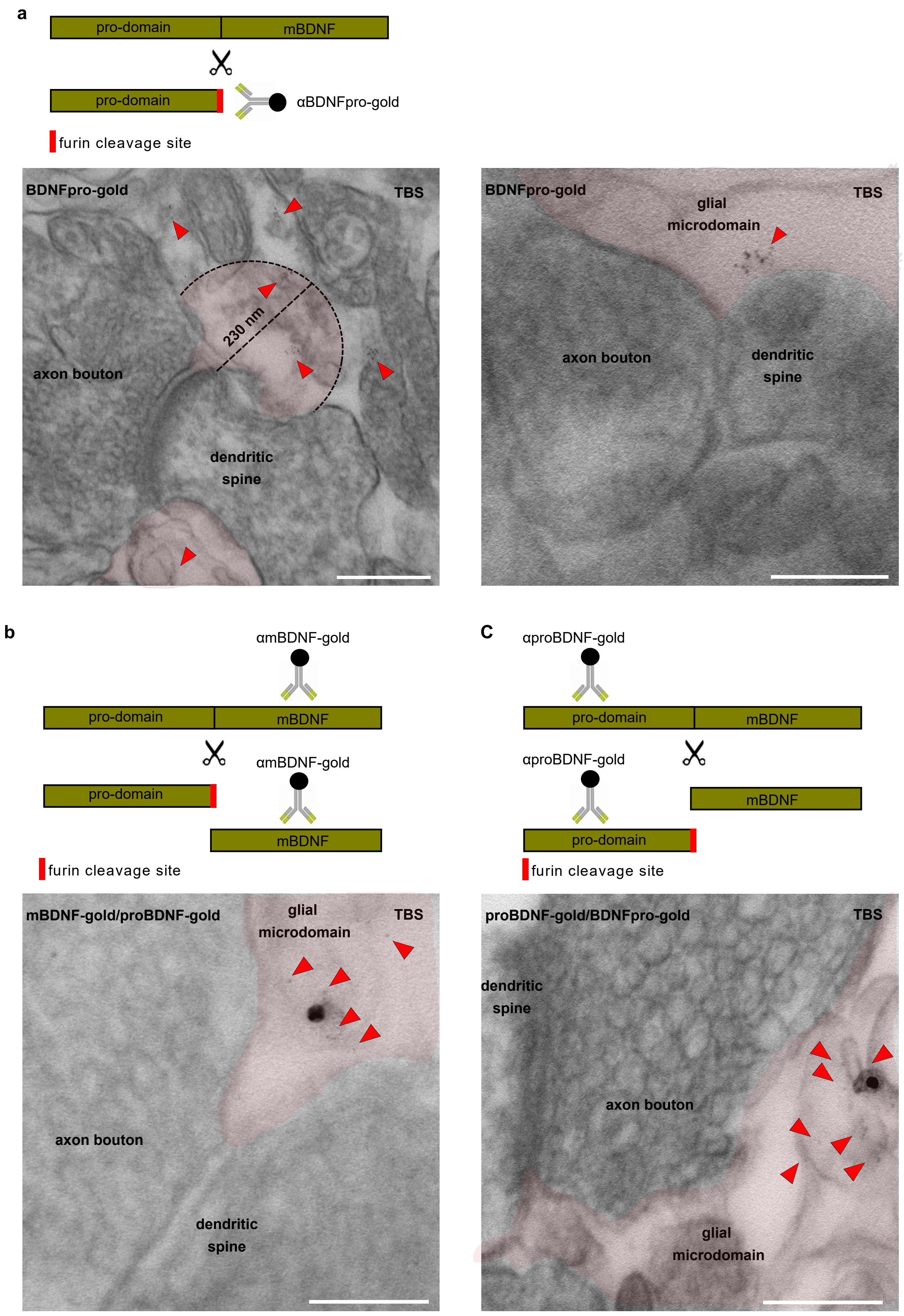

a
pro-domain
mBDNF
pro-domain
αBDNFpro-gold
furin cleavage site
BDNFpro-gold
TBS
230 nm
axon bouton
dendritic spine
BDNFpro-gold
TBS
glial microdomain
axon bouton
dendritic spine
b
αmBDNF-gold
pro-domain
mBDNF
αmBDNF-gold
pro-domain
mBDNF
furin cleavage site
C
αproBDNF-gold
pro-domain
mBDNF
αproBDNF-gold
mBDNF
pro-domain
furin cleavage site
mBDNF-gold/proBDNF-gold
glial microdomain
TBS
axon bouton
dendritic spine
proBDNF-gold/BDNFpro-gold
TBS
dendritic spine
axon bouton
glial microdomain

Supplementary Fig. 4: ultrastructural localization of neurotrophins in astrocytic microdomains

(a) Schematic representation of cleaved BDNFpro domain. αBDNFpro antibody recognizes the furin cleavage site of the prodomain. EM images depicting BDNFpro-gold particles (red arrowheads) in a limited fraction (230 nm radius) of the astrocyte surrounding the axon bouton. Scale bars: 200 nm.

(b) Schematic representation of the different neurotrophins isoforms recognized by αmBDNF antibody. EM image depicting αmBDNF-gold localization at astrocytic microdomains in sections from TBS- slices. Scale bar: 100 nm.

(c) Schematic representation of the different neurotrophins isoforms recognized by αproBDNF antibody. EM images depicting αproBDNF-gold localization at astrocytic microdomains in sections from TBS- slices. Scale bar: 100 nm.

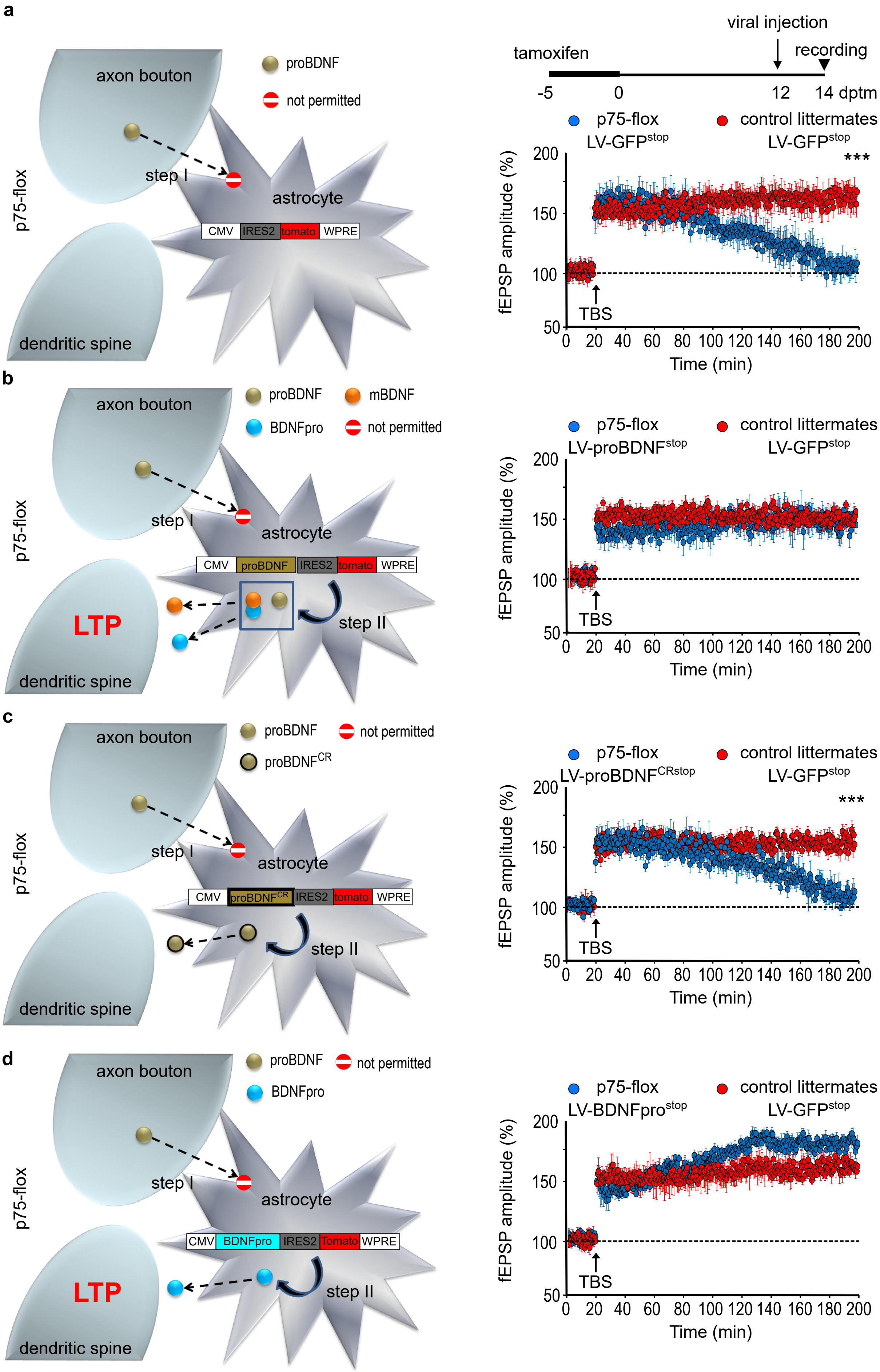
a
axon bouton
proBDNF
not permitted
step I
astrocyte
p75-flox
CMV IRES2 tomato WPRE
dendritic spine
viral injection
tamoxifen
recording
-5 0 12 14 dptm
p75-flox LV-GFPstop
control littermates LV-GFPstop
***
fEPSP amplitude (%)
TBS
Time (min)
b
axon bouton
proBDNF
mBDNF
BDNFpro
not permitted
step I
astrocyte
CMV proBDNF IRES2 tomato WPRE
LTP
step II
dendritic spine
p75-flox LV-proBDNFstop
control littermates LV-GFPstop
c
axon bouton
proBDNF
proBDNFCR
not permitted
step I
astrocyte
CMV proBDNFCR IRES2 tomato WPRE
step II
dendritic spine
p75-flox LV-proBDNFCRstop
control littermates LV-GFPstop
***
d
axon bouton
proBDNF
BDNFpro
not permitted
step I
astrocyte
CMV BDNFpro IRES2 Tomato WPRE
LTP
step II
dendritic spine
p75-flox LV-BDNFprostop
control littermates LV-GFPstop

Supplementary Fig.5: rescues of LTP deficit in p75-flox mice

(a) Schematic representation of the experimental design. Step I, deletion of $p75^{NTR}$ in astrocytes from tamoxifen-treated p75-flox mice precludes proBDNF transfer from neurons to astrocyte following TBS. LV-GFP^{stop} transduction in astrocytes results in loss of the GFP and expression of Tomato reporter. Graph shows LTP evoked in slices from p75-flox mice and control littermates treated with tamoxifen (-5 to 0) and injected with the appropriate lentivirus (12 dptm). *** $p<0.001$ (Unpaired t-test); (control littermates/LV-GFP^{stop} 166,57 ± 3,78 %, p75-flox/LV-GFP^{stop} 107,22 ± 7,16 % fEPSP 180 min from TBS; n = 7 slices, 5 mice for control littermates/LV-GFP^{stop}; n = 9 slices, 5 mice for p75-flox/LV-GFP^{stop}).

(b) Schematic representation of the experimental design. Step I, as in (a). Step II, LV-$proBDNF^{stop}$ transduction in astrocytes results in loss of the GFP and proBDNF expression. Graph shows LTP evoked in slices from p75-flox mice and control littermates as in (a). (control littermates/LV-GFP^{stop} 153,49 ± 2,22 %, p75-flox/LV-$proBDNF^{stop}$ 166,57 ± 3,78 fEPSP 180 min from TBS; (n = 6 slices, 5 mice for control littermates/LV-GFP^{stop}; n = 8 slices, 4 mice for p75-flox/LV-$proBDNF^{stop}$).

(c) Schematic representation of the experimental design. Step I, as in (a). Step II, LV-$proBDNF^{CRstop}$ transduction in astrocytes results in loss of the GFP and expression of the cleavage-resistant $proBDNF^{CR}$. Graph shows LTP evoked in slices from p75-flox mice and control littermates as in (a). *** $p<0.001$ (Unpaired t-test); (control littermates/LV-GFP^{stop} 149,72 ± 5,93 %, p75-flox/LV- $proBDNF^{CRstop}$ 109, 19 ± 7,00 % fEPSP 180 min from TBS; n = 6 slices, 4 mice for control littermates/LV-GFP^{stop}; n = 10 slices, 6 mice for p75-flox/LV-$proBDNF^{CRstop}$).

(d) Schematic representation of the experimental design. Step I, as in (a). Step II, LV-$BDNFpro^{stop}$ transduction in astrocytes results in loss of the GFP and expression of BDNFpro. Graph shows LTP evoked in slices from p75-flox mice and control littermates as in (a). Data are presented as mean ± SEM; (control littermates/LV-GFP^{stop} 159,19 ± 1,70 %, p75-flox/LV- $BDNFpro^{stop}$ 178,15 ± 9,84 % fEPSP 180 min from TBS; n = 7 slices, 6 mice for control littermates/LV-GFP^{stop}; n = 9 slices, 5 mice for p75-flox/LV-$BDNFpro^{stop}$).

Data are presented as mean ± SEM;

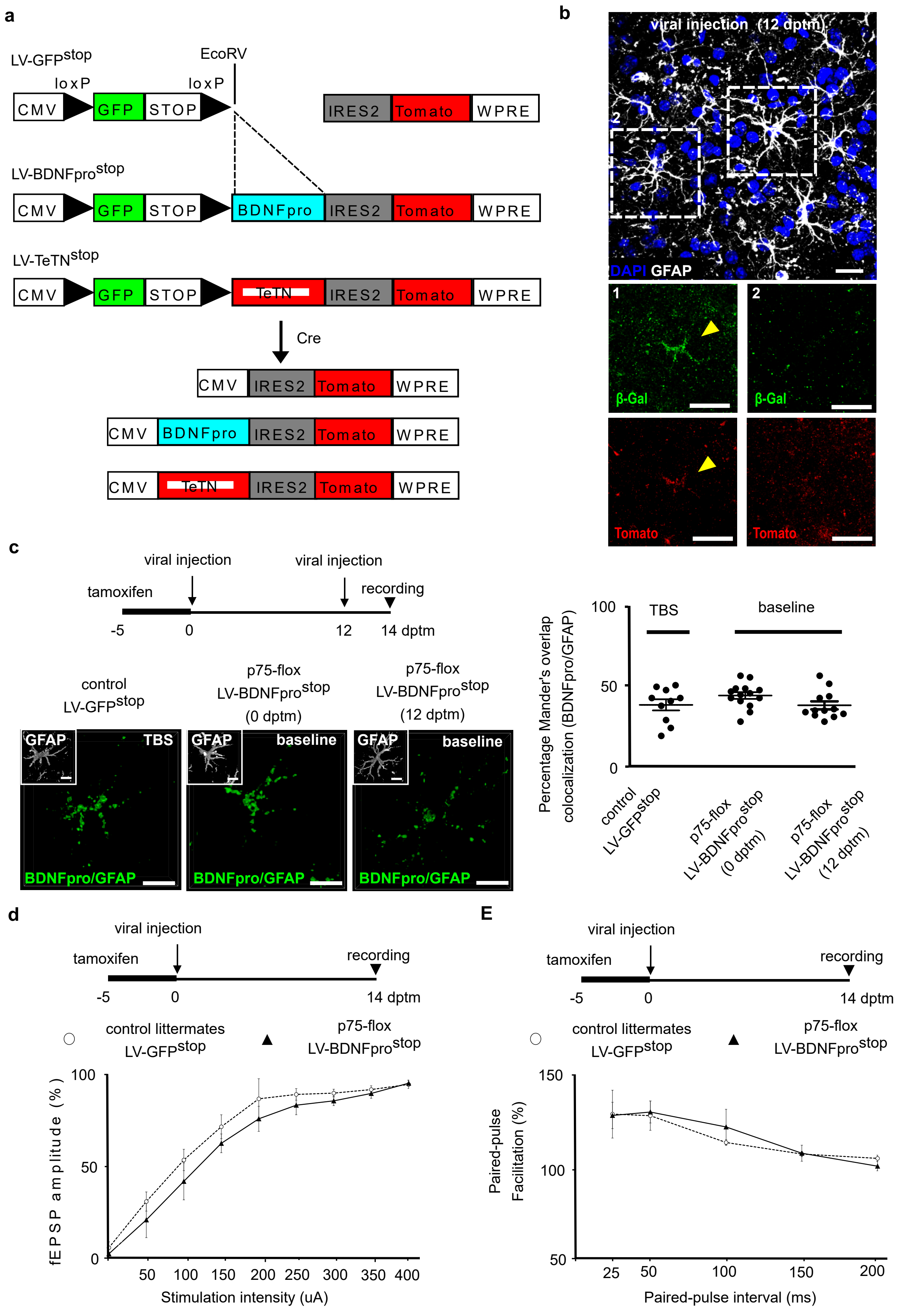
a
LV-GFPstop
EcoRV
loxP
loxP
CMV
GFP
STOP
IRES2
Tomato
WPRE
LV-BDNFprostop
BDNFpro
LV-TeTNstop
TeTN
Cre
b
viral injection (12 dptm)
DAPI GFAP
β-Gal
Tomato
c
tamoxifen
viral injection
viral injection
recording
-5
0
12
14 dptm
control
LV-GFPstop
p75-flox
LV-BDNFprostop
(0 dptm)
(12 dptm)
GFAP
TBS
baseline
BDNFpro/GFAP
Percentage Mander's overlap
colocalization (BDNFpro/GFAP)
100
50
0
d
recording
14 dptm
control littermates
fEPSP amplitude (%)
Stimulation intensity (uA)
50 100 150 200 250 300 350 400
E
Paired-pulse
Facilitation (%)
150
Paired-pulse interval (ms)
25 50 100 150 200

Supplementary Fig.6: transduction properties of lentiviral constructs

(a) Schematic overview of the lentiviral vectors. A CMV promoter drives GFP expression stopped with stop codon. Two loxP sites flank a gene cassette encoding GFP for Cre-recombination. Tomato is under control of the IRES2 site and followed by the WPRE element. A single blunt EcoRV site for cloning BDNFpro or TeTN is indicated. Cre-recombination in astrocytes results in loss of the GFP and expression of BDNFpro or TeTN.

(b) z-stack reconstruction shows cortical astrocytes labeled by GFAP in tamoxifen-induced mice) Cre-recombination (β-Gal+) results in selective expression of the virus reporter gene in astrocytes. Representative GFAP+/β-Gal+/Tomato+ (square 1) and GFAP+/β-Gal-/Tomato- (square 2) astrocytes are shown. Scale bars: 10 µm.

(c) Schematic overview of the experimental design. p75-flox mice were induced with tamoxifen for 5 days (-5 to 0) and injected with lentiviruses at 0 and 12 dptm. At 14 dptm cortical slices from both injection times were subjected to basal stimulation (baseline) and processed for immunodetection. z-stack reconstruction shows BDNFpro/GFAP colocalization in astrocytes from p75-flox mice injected with LV-BDNFpro^{stop} at 0 and 12 dptm. Slices from tamoxifen-treated control littermates injected with LV-GFP^{stop} (0 dptm) and stimulated with TBS are also shown. Insets show GFAP signal. Quantification of BDNFpro/GFAP colocalization signal using Mander's overlap is shown. (n = 10 cells, 4 slices, 3 mice for control littermates/LV-GFP^{stop}/TBS; n = 14 cells, 4 slices, 4 mice for p75-flox/LV-BDNFpro^{stop}/0 dptm/baseline; n = 12 cells, 4 slices, 4 mice for p75-flox/LV-BDNFpro^{stop}/12 dptm/baseline). Scale bars: 10 µm.

(d) Schematic diagram shows the experimental paradigm. Mice were induced with tamoxifen for 5 days (-5 to 0) and injected with LV-GFP^{stop} or LV-BDNFpro^{stop} (0 dptm) for field recording (14 dptm). Graph shows input\output curve (n=12 slices, 4 mice for control littermates/LV-GFP^{stop}; n=14 slices, 4 mice for p75-flox/LV-BDNFpro^{stop}).

(e) Schematic diagram shows the experimental paradigm as in (d). Graph shows paired-pulse facilitation with an interstimulus interval of 25, 50, 100, 150 and 200 ms. (n=12 slices, 4 mice for control littermates/LV-GFP^{stop}; n=14 slices, 4 mice for p75-flox/LV-BDNFpro^{stop}).

Data are presented as mean ± SEM;

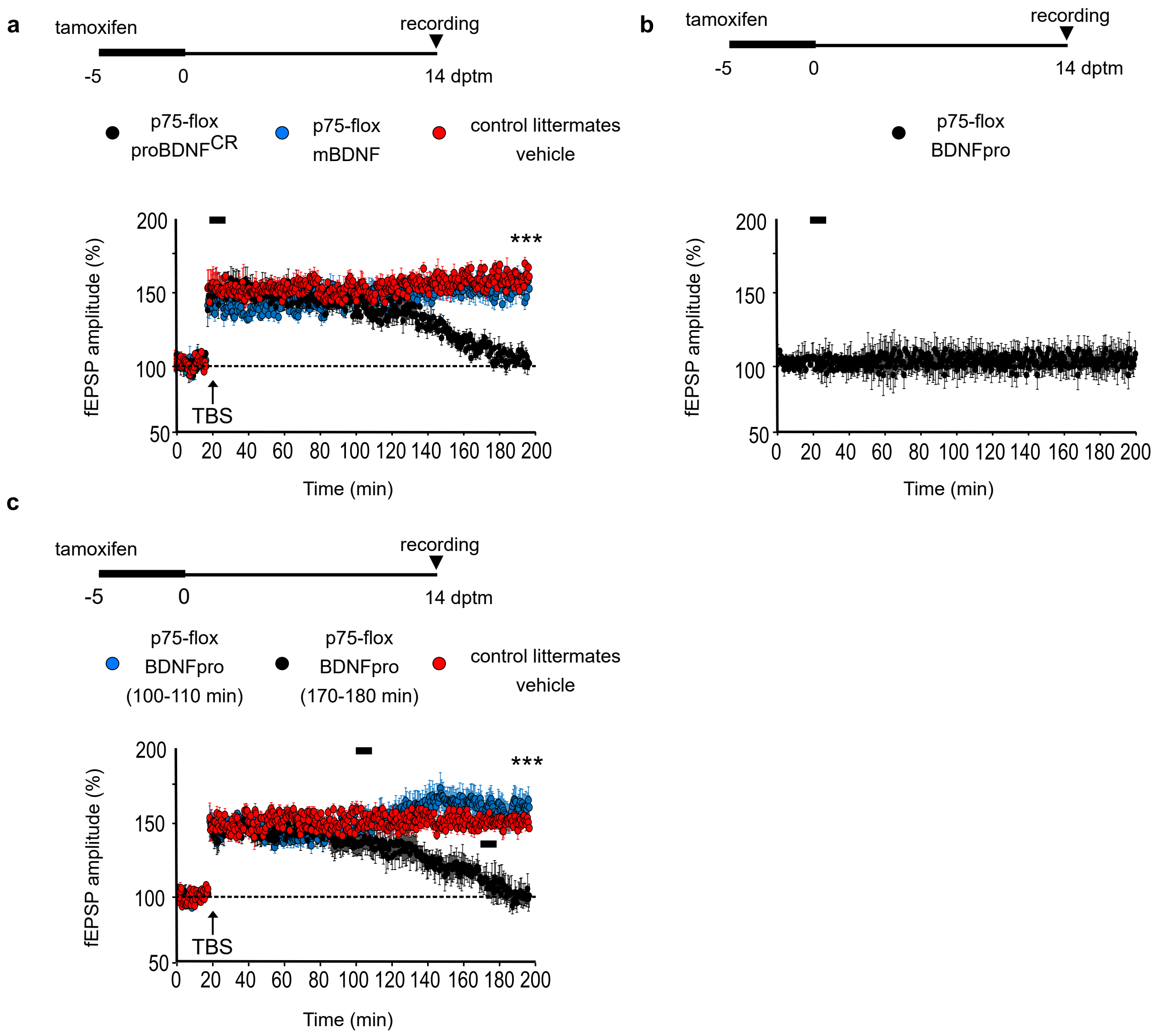

a
tamoxifen
recording
-5
0
14 dptm
p75-flox proBDNF^CR
p75-flox mBDNF
control littermates vehicle
fEPSP amplitude (%)
200
150
100
50
***
TBS
0 20 40 60 80 100 120 140 160 180 200
Time (min)
b
tamoxifen
recording
-5
0
14 dptm
p75-flox BDNFpro
fEPSP amplitude (%)
200
150
100
50
0 20 40 60 80 100 120 140 160 180 200
Time (min)
c
tamoxifen
recording
-5
0
14 dptm
p75-flox BDNFpro (100-110 min)
p75-flox BDNFpro (170-180 min)
control littermates vehicle
fEPSP amplitude (%)
200
150
100
50
***
TBS
0 20 40 60 80 100 120 140 160 180 200
Time (min)

Supplementary Fig.7: exogenous neurotrophins rescue LTP deficit in p75-flox mice

(a) Schematic diagram shows the experimental paradigm. Tamoxifen was given for 5 consecutive days (-5 to 0) and recording performed at 14 dptm. Histogram depicts LTP evoked in slices from p75-flox mice and control littermates perfused (18 to 28 min) with vehicle or exogenous mBDNF (10 ng/ml) or proBDNF^{CR} (20 ng/ml). *** p<0.001 (Unpaired t-test); (p75-flox/proBDNF^{CR} 100,40 ± 4,25 %, p75-flox/mBDNF 150,66 ± 5,27 % and control littermates/vehicle 158,69 ± 2,30 fEPSP 180 min from TBS; n=8 slices, 4 mice for p75-flox/proBDNF^{CR}; n=9 slices, 5 mice for p75-flox/mBDNF; n=7 slices, 4 mice for control littermates/vehicle).

(b) Schematic diagram shows the experimental paradigm as in (a). Histogram depicts BDNFpro (10 ng/ml) treatment (18 to 28 min) in the absence of TBS-stimulation. (n=6 slices, 3 mice).

(c) Schematic diagram shows the experimental paradigm as in (a). Histogram depicts LTP evoked in slices from p75-flox mice and control littermates perfused with exogenous BDNFpro (10 ng/ml) or vehicle at different times (100 to 110 min; 170 to 180 min). *** p<0.001 (Unpaired t-test); (p75-flox/BDNFpro (100-110 min) 160,07 ± 7,92 %, p75-flox/BDNFpro (170-180 min) 101,56 ± 7,79 % and control littermates/vehicle 146,13 ± 3,30 fEPSP 180 min from TBS; (n=6 slices, 3 mice for p75-flox/BDNFpro (100-110 min); n=7 slices, 4 mice for p75-flox/BDNFpro (170-180 min); n= 6 slices, 4 mice for control littermates/vehicle).

Data are presented as mean ±SEM;

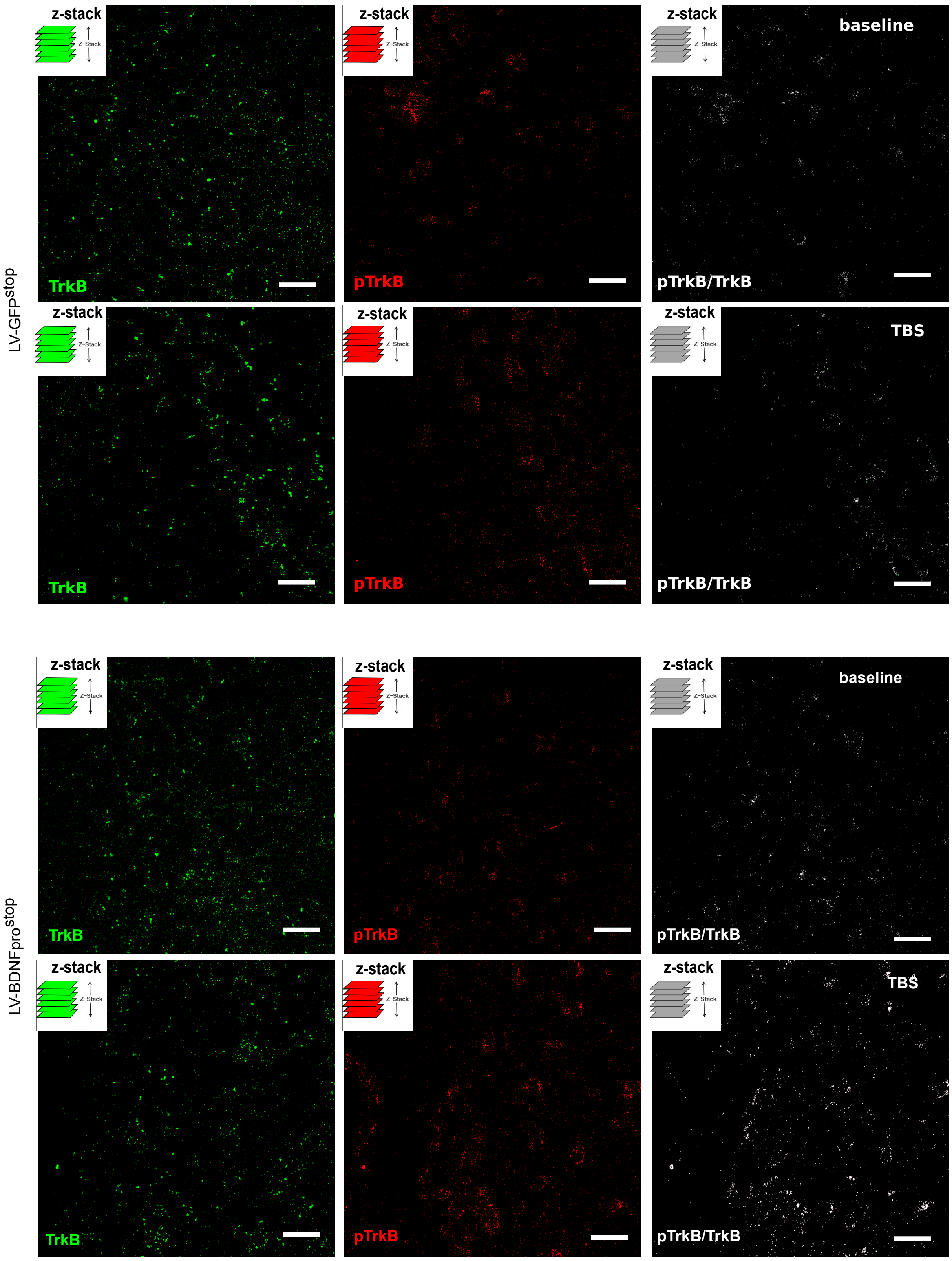
LV-GFP^{stop}
LV-BDNF$_{pro}$^{stop}
z-stack
Z-Stack
baseline
TBS
TrkB
pTrkB
pTrkB/TrkB

Supplementary Fig.8: TrkB/pTrkB colocalization

Confocal images of TrkB, pTrkB and TrkB/pTrkB colocalization signals in baseline- and TBS-slices from p75-flox mice injected with LV-GFP^{stop} and LV-BDNFpro^{stop}. Scale bars: 40 μm.
