# Supplementary material for: Astrocytic microdomains from mouse cortex gain molecular control over long-term information storage and memory retention
Source: Commun Biol. 2021 Oct 5;4:1152. doi: 10.1038/s42003-021-02678-x (PMC8492720; doi:10.1038/s42003-021-02678-x)
Supplement: Supplementary file 3 — Description of Additional Supplementary Files [file 42003_2021_2678_MOESM3_ESM.pdf]

Description of Additional Supplementary Files

File name: Supplementary Data 1.
Description: Source data underlying Figs. 1, 3 and 5 -8.
