# Supplementary material for: Astrocytic microdomains from mouse cortex gain molecular control over long-term information storage and memory retention
Source: Commun Biol. 2021 Oct 5;4:1152. doi: 10.1038/s42003-021-02678-x (PMC8492720; doi:10.1038/s42003-021-02678-x)
Supplement: Supplementary file 5 — Reporting Summary [file 42003_2021_2678_MOESM5_ESM.pdf]

nature research

Corresponding author(s): Marco Canossa
Beatrice Vignoli

Last updated by author(s): Aug 31, 2021

Reporting Summary

Nature Research wishes to improve the reproducibility of the work that we publish. This form provides structure for consistency and transparency in reporting. For further information on Nature Research policies, see our Editorial Policies and the Editorial Policy Checklist.

Statistics

For all statistical analyses, confirm that the following items are present in the figure legend, table legend, main text, or Methods section.

n/a	Confirmed	
☐	☒	The exact sample size (*n*) for each experimental group/condition, given as a discrete number and unit of measurement
☐	☒	A statement on whether measurements were taken from distinct samples or whether the same sample was measured repeatedly
☐	☒	The statistical test(s) used AND whether they are one- or two-sided<br>*Only common tests should be described solely by name; describe more complex techniques in the Methods section.*
☒	☐	A description of all covariates tested
☐	☒	A description of any assumptions or corrections, such as tests of normality and adjustment for multiple comparisons
☐	☒	A full description of the statistical parameters including central tendency (e.g. means) or other basic estimates (e.g. regression coefficient) AND variation (e.g. standard deviation) or associated estimates of uncertainty (e.g. confidence intervals)
☐	☒	For null hypothesis testing, the test statistic (e.g. *F*, *t*, *r*) with confidence intervals, effect sizes, degrees of freedom and *P* value noted<br>*Give P values as exact values whenever suitable.*
☒	☐	For Bayesian analysis, information on the choice of priors and Markov chain Monte Carlo settings
☒	☐	For hierarchical and complex designs, identification of the appropriate level for tests and full reporting of outcomes
☒	☐	Estimates of effect sizes (e.g. Cohen's *d*, Pearson's *r*), indicating how they were calculated

Our web collection on statistics for biologists contains articles on many of the points above.

Software and code

Policy information about availability of computer code

Data collection	For imaging experiments data collection were performed with NIS-Element software (Nikon). For electrophysiological experiments data collection were performed using Axonscope 8.0 software (Molecular Devices). For behavioral experiments data collection were performed with EthoVision software (Noldus). For western blot experiments data collection were performed with BioRad Chemidoc MP Imaging System.
Data analysis	Data analysis were performed with Nis Element software (Nikon), Prism software (GraphPad), Excel (Microsoft Office), Clampfit 10.6 software (Molecular Device), EthoVision software (Noldus), Image J (NIH).

For manuscripts utilizing custom algorithms or software that are central to the research but not yet described in published literature, software must be made available to editors and reviewers. We strongly encourage code deposition in a community repository (e.g. GitHub). See the Nature Research guidelines for submitting code & software for further information.

Data

Policy information about availability of data

All manuscripts must include a data availability statement. This statement should provide the following information, where applicable:
- Accession codes, unique identifiers, or web links for publicly available datasets
- A list of figures that have associated raw data
- A description of any restrictions on data availability

The authors declare that all data supporting the findings of this study are available within the paper and its supplementary information files.

Field-specific reporting

Please select the one below that is the best fit for your research. If you are not sure, read the appropriate sections before making your selection.

☒ Life sciences ☐ Behavioural & social sciences ☐ Ecological, evolutionary & environmental sciences

For a reference copy of the document with all sections, see nature.com/documents/nr-reporting-summary-flat.pdf

Life sciences study design

All studies must disclose on these points even when the disclosure is negative.

Sample size	We indicated the sample size (n) of each experiment in the figure caption in the main manuscript and supplementary information file. Sample size were chosen to support meaningful conclusions.
Data exclusions	Statistical analysis included all the data point obtained, with the exception of experiments in which negative and/or positive controls did not give the expected outcome, which were discarded.
Replication	All experiments were replicated at least three times, unless indicated differently.
Randomization	We did not randomize the samples.
Blinding	All data were collected and analyzed blindly.

Reporting for specific materials, systems and methods

We require information from authors about some types of materials, experimental systems and methods used in many studies. Here, indicate whether each material, system or method listed is relevant to your study. If you are not sure if a list item applies to your research, read the appropriate section before selecting a response.

Materials & experimental systems

n/a	Involved in the study	
☐	☒	Antibodies
☒	☐	Eukaryotic cell lines
☒	☐	Palaeontology and archaeology
☐	☒	Animals and other organisms
☒	☐	Human research participants
☒	☐	Clinical data
☒	☐	Dual use research of concern

Methods

n/a	Involved in the study	
☒	☐	ChIP-seq
☒	☐	Flow cytometry
☒	☐	MRI-based neuroimaging

Antibodies

Antibodies used	The following antibodies were used: rabbit α-GFP (Thermo Fisher Scientific Cat#A-6455; RRID:AB#2536208; IHC 1:1000 ICC 1:1000), chicken α-GFP (Thermo Fisher Scientific Cat#A10262; RRID:AB#2534023, IHC 1:1000), chicken α-BDNF (Promega Cat#G1641; RRID:AB#430850, IHC 1:300; WB 1:500), rabbit α-BDNF (Alomone Labs Cat# ANT-010; RRID:AB_2039756, EM 1:20), chicken α-proBDNF (Millipore Cat#AB9042; RID:AB#2274709, IHC 1:300), rabbit α-proBDNF (Alomone Labs Cat#ANT-006; RRID:AB_2039758, EM 1:20), rabbit α-BDNFpro (Laboratory of Bai Lu, Govern Institute for Brain Research, Tsinghua University, Beijing, IHC 1:300, EM 1:20, WB 1:500), mouse α-NeuN (Abcam Cat#ab77315; RRID:AB#1566475, IHC 1:1000), guinea pig α-NeuN (Millipore Cat#ABN90; RRID:AB#11205592, IHC 1:1000), rabbit α-pTrkB (Tyr 816) (Laboratory of Moses Chao, Skirball Intitute of Biomolecular Medicine, New York, USA, IHC 1,25 mg/ml), goat α-TrkB (Santa Cruz Biotechnology Cat#sc-12-G; RRID:AB#632558, IHC 1:300), mouse α-PSD95 (Merck-Millipore Cat#MAB1596; RRID:AB_2092365, IHC 1:500), sheep α-SorCS2 (R&D system Cat#AF4238; RRID:AB_10645642), rabbit α -SorCS2 (MyBioSource Cat# MBS5302436), rabbit α-p75NTR (Promega Cat#G3231; RRID:AB_430853, IHC 1:1000), chicken α-GFAP (Abcam Cat#ab134436; RRID:AB_2818977, IHC 1:1000), mouse α-GFAP (Abcam Cat#ab10062; RRID:AB_296804, IHC 1:1000), mouse α-synaptobrevin2\VAMP2 (Synaptic System Cat#104 211; RRID:AB_2619758, IHC 1:300), rabbit α-beta galactosidase (Proteintech Cat#15518-1-AP; RRID:AB_2263448, IHC 1:500), rabbit α -RFP (Rockland Antibodies Cat#600-401-379; RRID:AB_2209751 IHC 1:1000), mouse α DsRed (Santa Cruz Biotechnology Cat# sc-390909; RRID:AB_2801575 IHC 1:1000).
Validation	Specificity of rabbit α-BDNFpro (kindly provided by Bai Lu) were validated using western blot analysis on recombinant proteins as reported in the manuscript.<br>The rest of reported antibodies were extensively been validated in previous publication, appropriately referenced into the manuscript.

Animals and other organisms

Policy information about studies involving animals; ARRIVE guidelines recommended for reporting animal research

Laboratory animals	In this study the following laboratory animals were used:<br>- p75-flox mice (generated by crossing loxP-p75NTR-loxP mice kindly provided by B. Pierchala, University of Michigan School of Dentistry, USA with GLAST-CreERT2 Rosa-CAGloxP-stop-loxP(LSL)-R26R mice kindly provided by Prof. M Gotz, LMU, Munich, Germany): Mus musculus, C57Bl6, male, age P35-50;<br>- GFAP-GFP mice (kindly provided by Prof. A. Buffo, NICO, Torino, Italy): Mus musculus, FVB/N, male, 2 month of age.
Wild animals	Wild animals were not used in this study.
Field-collected samples	This study did not involve sample collected from the field.
Ethics oversight	All experiments were performed in accordance with European Union guidelines as approved by the institutional animal care and utilization committee (authorizations n°507/2017-PR; 76/2020 PR).

Note that full information on the approval of the study protocol must also be provided in the manuscript.
